# The Book World of Medicine and Science

**Published:** 1893-04-29

**Authors:** 


					Vlll
THE HOSPITAL. April 29,1893.
The Book World of Medicine and Science.
BOOK REVIEWS.
Elementary Physiology. By Alfred Schofield, M.D.,
M.R.C.S., author of "Physiology for Sohooln," "A
Manual of Hygiene," &c. (Cassell and Co. : London,
Paris, and Melbourne.)
Dr. Schofield does not only know physiology, he knows
what students wish to know about it, and how to give them
that knowledge clearly and concisely. While this volume
by no means pretends to take the place of the larger text-
books, it will in all probability form, to the average man, a
better introduction to the science than any of them, because
it is so completely free from mere theory or excessive detail.
To a youth plunging, sometimes without adequate guidanoe,
into a new subject, nothing is more confusing than too full a
text book. He cannot judge the respective importance of
various statements, and his mind becomes filled with a con-
fusion of facts, theories, and experiments, which at the
moment of examination iB apt to leave his brain a blank.
We by no means say that Dr. Schofield's book contains all
that a medical man ought to know of physiology, and that
in the course of his studies he need not go on to any more
advanced and elaborate text-book, but we are strongly of
opinion that in order to get full value out of such a text-
book he ought to have the outlines of physiology fixed in hia
mind by a clear and compendious work like this. It is not,
we think, to Dr. Schofield's discredit that he does not
disdain to impress a fact in his reader's mind by means of
such a " tip " as this : " The liver has five letters, five lobes,
five ligaments, five fissures, five vessels, five functions, and
weighs fifty ounces." Factu thus impressed are likely to
stick, and this is the main object. The diagrams are admir>
ably, clear, and the coloured plates should be especially
useful in aiding the beginner in microscopic investigation.
Altogether, we recommend this volume as a handy, clear,
and practical introduction to the study of physiology,
April 29, 1893. THE HOSPITAL. ix
THE BOOK WORLD OF MEDICINE AND SCIENCE.-(continued).
surgical treatment of hernia. He has a method of his own
for the radical cure by whioh he seeks to strengthen the
internal ring, either by the inverted neck of the sac or by a
pad or omentum. All such methods are faulty in concep-
tion ; the true ideal is to establish the valve-like obliquity of
the iDguinal canal. The arguments for and against the
radical cure are fairly stated, and all through the book we
find evidence of the experienced surgeon and good observer
as well as keen ins tructor.
HEALTH RESORTS.
Margate as a Health Resort. By H. Evelyn
Crook, M.D., B.S., F.R.O.S., Eng.
Margate rightly holds a very high place as a health resort,
and in this little pamphlet, Dr. Crook has stated the facts
as to its climate, sanitation, and vital statistics on which
an opinion of its value in preventing and curing disease may
be based. Useful for reference.
Orotova as a Health Resort. By George Victor Percy,
M.B., M.R.C.S. (London : Chas. J. Clark. 1893.)
New health resorts are being continually discovered, and
one of the latest additions to a long list is the Canary
Islands. Of these Teneriffe has high claims to preference,
and Orotava appears to be the most favoured spot on that
island. Dr. Percy gives a graphic description of its situa-
tion, scenery, and climate, and his readers will be very
strongly tempted to teBt the truth of his statements when
convalescing from illness, or jaded and overworked.
MEDICAL JOURNALS : BRITISH AND FOREIGN.
The Clinical Journal. A Weekly Record of Clinical
Medicine and Surgery, with their Special Branches.
We are glad to find that this new venture in medical
journalism is thoroughly maintaining its interest. The
clinical lectures are well reported, are very varied in their
range of subject, and are taken from a large number of con-
tributors. '1'he form, size, and type of the journal are
alike convenient, and we recommend our readers to Bee it
regularly, and thus easily and pleasantly keep abreast of the
teaching in our medioal schools.
La Riforma Medica.
We have received three numbers of this journal, whioh is
published every week in Naples under the editorship of
Professor G. Bummo. It contains original articles on
medical and surgical topics, reports of societies, and an
editorial article. Among others we find a clinical lecture of
Dr. Goodhart's on " Cheyne-Stokes' Respiration."
The Edinburgh Medical Journal for April is a fair
average number ; if none of the original articles are of strik-
ing excellence or importance, they deal with matters of
importance and interest. Professor Simpson writes on the
induotion of labour by the intrauterine injection of glycerine;
Dr. Wyllie on the Disorders of Speech : Pachydermia of the
Larynx. Addison's Disease, Uterine Rotation, and Bleeding
from the Urachus are other topics.
The Dublin Journal of Medical Science for April is a
good number. Dr. Smyly reports 112 consecutive
abdominal sections performed in the Rotunda Hospital?a
very interesting series. Mr. Doyle writes on two cases of
disease of the mastoid and detrous bone. The reports of the
meetings of the Academy of Medioine are well done, and
include a brief reference to a unique case, a successful case of
ligature of the innominate artery, by Mr. Coppinger.
The Bristol Medico-Chirurgical Journal for March
opens with a bright article by Dr. Kent Spendes, describing
his own experiences when attacked with acute inflammation
around the upper cervical vertebra. A successful case of
operation for extra-uterine gestation, and a paper on
"Pleural Effusions," complete the original matter. Reviews,
articles on the Progress of Medicine, &c., complete an
interesting number.

				

